# The Association Between Social Support and Mental Health-Related Quality of Life Among Older Adults in Puerto Rico

**DOI:** 10.1093/geroni/igaf122.1503

**Published:** 2025-12-31

**Authors:** Ebenezer Martey, Maricruz Rivera-Hernandez, Ross Andel, Olivio Clay, Ana Davila, Michael Crowe

**Affiliations:** The University of Alabama at Birmingham, Birmingham, Alabama, United States; Brown University School of Public Health, Providence, Rhode Island, United States; Arizona State University, Arizona State University, Arizona, United States; The University of Alabama at Birmingham, Birmingham, Alabama, United States; University of Puerto Rico, San Juan, Puerto Rico, United States; The University of Alabama at Birmingham, Birmingham, Alabama, United States

## Abstract

Emigration is a major concern for Puerto Rico residents as they lose younger people to better economic opportunities on the US mainland. This has probably led to reduced caregiving availability and social support for older adults, but limited research exists on how social support and sociodemographic factors are related to quality of life (QoL) among older Puerto Ricans. We examined the relationship between social support and mental health-related QoL using data from a long-term follow-up of the Puerto Rican Elder Health Conditions (PREHCO) Study (Wave 3; n = 617). QoL was measured with the Veterans RAND 12-Item Health Survey (VR-12) mental component summary. Social support network (SSN) was assessed using the 6-item Lubben Social Network Scale. Multiple regression was used to examine associations between QoL and social support while adjusting for age, sex, education and living alone. The average age of participants was 84 years and 63% were women. Participants had an average of 9.2 years of education and 37% were living alone. SSN was positively associated with education but not with other demographic factors. QoL was higher among women but showed no significant associations with other demographic factors. In the multivariable regression model, stronger SSN was associated with higher QoL score (β = 0.11, p = 0.006). The association between QoL and SSN was similar in people who lived alone versus not alone. These findings suggest the importance of maintaining a strong SSN as it appears to play a significant role in the mental health-related QoL of older adults in Puerto Rico.

